# Pancreatic Cancer: Challenges and Opportunities in Locoregional Therapies

**DOI:** 10.3390/cancers14174257

**Published:** 2022-08-31

**Authors:** Alaa Y. Bazeed, Candace M. Day, Sanjay Garg

**Affiliations:** Centre for Pharmaceutical Innovation, University of South Australia, North Terrace, Adelaide, SA 5000, Australia

**Keywords:** pancreatic ductal adenocarcinoma, localised therapy, drug delivery, thermal ablation, irreversible electroporation, stereotactic body radiotherapy, intra-arterial infusion chemotherapy, isolated upper abdominal perfusion, photodynamic therapy

## Abstract

**Simple Summary:**

Pancreatic cancer is a serious ongoing global health burden, with an overall 5-year survival rate of less than 5%. One major hurdle in the treatment of this disease is the predominantly elderly patient population, leading to their ineligibility for curative surgery and a low rate of successful outcomes. Systemic administration introduces chemo-agents throughout the body via the blood, attacking not only tumours but also healthy organs. When localised interventions are employed, chemo-agents are retained specifically at tumour site, minimizing unwanted toxicity. As a result, there is a growing interest in finding novel localised interventions as alternatives to systemic therapy. Here, we present a detailed review of current locoregional therapies used in pancreatic cancer therapy. This work aims to present a thorough guide for researchers and clinicians intended to employ established and novel localised interventions in the treatment of pancreatic cancer. Furthermore, we present our insights and opinions on the potential ideals to improve these tools.

**Abstract:**

Pancreatic cancer (PC) remains the seventh leading cause of cancer-related deaths worldwide and the third in the United States, making it one of the most lethal solid malignancies. Unfortunately, the symptoms of this disease are not very apparent despite an increasing incidence rate. Therefore, at the time of diagnosis, 45% of patients have already developed metastatic tumours. Due to the aggressive nature of the pancreatic tumours, local interventions are required in addition to first-line treatments. Locoregional interventions affect a specific area of the pancreas to minimize local tumour recurrence and reduce the side effects on surrounding healthy tissues. However, compared to the number of new studies on systemic therapy, very little research has been conducted on localised interventions for PC. To address this unbalanced focus and to shed light on the tremendous potentials of locoregional therapies, this work will provide a detailed discussion of various localised treatment strategies. Most importantly, to the best of our knowledge, the aspect of localised drug delivery systems used in PC was unprecedentedly discussed in this work. This review is meant for researchers and clinicians considering utilizing local therapy for the effective treatment of PC, providing a thorough guide on recent advancements in research and clinical trials toward locoregional interventions, together with the authors’ insight into their potential improvements.

## 1. Introduction

Pancreatic cancer (PC) is considered one of the most lethal solid malignancies due to its clinically silent and aggressive nature, making it an ongoing global health burden, with an overall 5-year survival rate of less than 5% [1]. With an increasing incidence and a parallel increase in death rate, PC is predicted to be the second leading cause of cancer-related deaths worldwide by the year 2030. Based on the United European Gastroenterology, PC is Europe’s deadliest cancer, killing over 95,000 patients every year, resulting in a life span of 5 months post-diagnosis [2,3]. Along with lung cancer, the United States Congress has declared pancreatic ductal adenocarcinoma (PDAC) as a “recalcitrant cancer” [4], while European Commission called it “neglected cancer”. Despite the devastating outcomes of this disease, research on PC has been the least funded among all cancers over the years, with less than 2% of the overall cancer funding in Europe [3]. At the time of the diagnosis, 35% of patients have developed locally advanced PDAC and 45% are metastatic [5]. Due to a lack of early detection and screening tools, it is advisable to actively prevent the risk of PC. This could be done through smoking cessation and maintaining a healthy lifestyle and body weight with balanced physical activities [6].

The first-line treatments for pancreatic tumours include surgery, chemotherapy, radiotherapy, and/or a combination of these modalities. Despite the recent advancement of the aforementioned treatment approaches, the prognosis and outcomes for the patients diagnosed with PC have remained very poor in the last 40–50 years. Even though surgery is still the primary treatment for the majority of cancers, most PCs are unresectable due to the late diagnosis, aggressive microenvironment, and the complex dense stromal compartment with deprived vascularization renders pancreatic tumour inaccessible to systemic therapies [7]. Due to the aggressive nature of the pancreatic tumour, multimodal therapies including resection, chemotherapy, and local interventions are required, to prevent the local recurrence of PC. Locoregional interventions involve various minimally invasive therapeutic procedures given as adjuvant therapy, in an attempt to improve the overall survival rate.

Acquiring a thorough knowledge of a disease is essential to developing effective treatments. As a result, this review will begin with an overview of epidemiology, risk factors, pathological hallmarks, and the current therapeutic strategies for PC. A detailed discussion on locoregional interventions other than surgery and their current advancement in the treatment of PC will then follow. 

## 2. Overview of Pancreatic Cancer

### 2.1. Epidemiology

In the year 2020, 495,773 people were diagnosed with PC globally, out of which 466,003 people did not survive [8]. Based on the American Cancer Society Report, PC is responsible for 3% of cancer deaths in the United States and 7% in the world [9]. In addition to this, more than 60,000 people are expected to be diagnosed with a pancreatic tumour in 2022, with more than half projected to be men, and 80% are projected to die within months post-diagnosis. Moreover, in Australia, the estimated number of PC cases diagnosed per year has doubled in the last 20 years [10]. Indeed, the incidence rates of PC have gone up by around 1% each year since 2000 in the U.S [11]. 

PC incidence is higher in males than in females. The mortality rate was 5.3 and cumulative risk was 0.62% in men, compared to 3.8, and 0.41% in women, respectively [8]. Moreover, PC is an age-related cancer where lower incidence is observed in the population under the age of 40, while the average diagnosis age is 70 years old [12]. Interestingly, high-income countries record a four-fold higher incidence rate, with Europe, the Americas, Australia, New Zealand, and Asia taking the lead compared to low-income countries. This could be attributed to excessive alcohol consumption, smoking, and diabetes, in addition to a longer life span and better access to diagnostic services [8].

### 2.2. Risk Factors 

#### 2.2.1. Smoking (Tobacco)

The significant association between cigarettes and increased risk of PC has been well-established through a large body of literature. Tobacco is believed to account for about 20% of PC cases, as the cancer risk increases in proportion with the daily number of cigarettes consumed and the duration of smoking [13]. More importantly, smoking accounts for 14% of the population attributed risk (PAR) compared to other risk factors such as alcohol abuse, body mass index, food, and physical exercise [14]. A genome study of 7937 pancreatic ductal adenocarcinoma (PDAC) cases in 2021 reported a genomic locus that significantly increased PDAC risk with smoking [15]. Furthermore, multiple studies have proven that smokers are at double the risk for PC compared to non-smokers [16,17]. For instance, in the International PC Cohort Consortium control study conducted in 2009, smokers with increasing intensity and period of cigarette abuse showed a higher risk of developing PC, compared to non-smokers [17]. In another meta-analysis study in 2,517,623 patients, smokers were reported to have a 40% greater risk compared to non-smokers [18].

#### 2.2.2. Diabetes

The association of diabetes with PC is complex, and a detailed conclusive picture has not been constructed. Several studies have suggested that diabetes is a risk factor for PC, while others have concluded that PDAC is a reason for patients to develop diabetes for unknown reasons [19]. Diabetic patients are at a three-fold higher risk of developing PDAC, compared to other cancers. Besides this, the highest risk PC is observed in the first two years of being diagnosed with diabetes and decreases as diabetes progresses [20].

#### 2.2.3. Body-Mass Index (BMI)

A large body of literature has also pointed to the higher risk of developing multiple cancers, including PC, with increased BMI. Individuals with BMI greater than 30 are at a 20% greater risk of developing PC, especially those carrying additional adipose tissues in the waistline area [21], in addition to an increased mortality rate [22]. In the older population, it was reported that having an increased BMI before the age of 50 strongly elevates the risk of PC [23].

#### 2.2.4. Alcohol Consumption 

It was reported that a liquor intake of more than three drinks a day could increase the risk of PC, and this risk is even higher when associated with smoking [20,24]. A suggested mechanism and explanation for the relation between alcohol and PDAC has been proposed, where the by-product of alcohol—acetaldehyde—can bind to DNA repair proteins, causing DNA damage and tumorigenesis [24]. 

#### 2.2.5. Pancreatitis

Chronic pancreatitis can be caused by cystic fibrosis, hereditary diseases, smoking, and alcohol abuse. Chronic pancreatitis is associated with a high risk of PC, as almost 5% of chronic pancreatitis patients develop PDAC in 20 years’ time from diagnosis [25]. On the other hand, pancreatitis is also considered a complication caused by PC [26]. 

#### 2.2.6. Family History and Genetics

Although nearly 10% of PC are hereditary, the mutations that occur in a person’s lifetime are more accountable for most PC cases. Having close relatives with PC increases the risk by 1.5–13-fold [12]. PC is highly heterogenous with more than 50% of patients presenting with somatic mutations in KRAS, TP53, SMAD4, and CDKN2A [27]. A higher risk of PC amongst family members is also observed when there is a family history of other cancers, such as breast, colon, and melanoma. Therefore, it is highly recommended that individuals having a family history of PC undergo genetic testing, and enrol themselves in surveillance programs with yearly endoscopic guided and magnetic resonance imaging [6].

### 2.3. Types and Tissue Architecture of PC

PCs can be classified into epithelial and non-epithelial, according to histological differentiation. Epithelial PCs are either exocrine or neuroendocrine [28]. More than 90% of the diagnosed PCs are pancreatic adenocarcinoma which occurs in the lining of the pancreatic duct [29]. Therefore, it is considered part of the exocrine PC. Other types of exocrine PCs include squamous, adenosquamous, and colloid carcinoma. Meanwhile, neuroendocrine PCs represent less than 5% of total PCs, which originates in the endocrine cells which are responsible for generating hormones such as insulin and glucagon [30]. 

**Figure 1:**
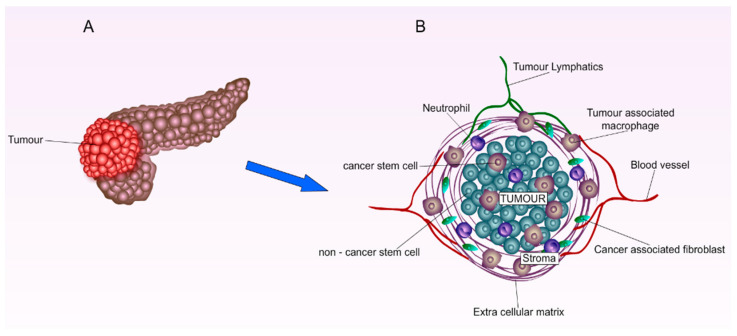
Graphical illustration of pancreatic tumour microenvironment. (**A**) Primary pancreatic tumour. (**B**) Enlarged pancreatic tumour environment: The cancerous cell cytokines induce the production of fibroblasts and stellate cells, leading to the build-up of the dense stroma compartment. The hostile dense and stromal structure increases intra-tumoral pressure, causing the hypo-vascularization or the collapse of blood vessels surrounding tumours. Due to the lack of a cancerous blood vessel matrix, EPR effects cannot be utilised efficiently for the delivery of systemic therapeutics.

The PC microenvironment is characterised by dense stromal structure, hypo-vascularization, and hypoxia which plays a major role in carcinogenesis, therapeutic extrinsic resistance, and disease progression (Figure 1) [31]. The stromal compartments account for 70% of the pancreatic tumour abundant with fibroblasts and stellate cells which are stimulated by cancerous cells cytokines to increase the stromal deposition of collagen and hyaluronic acid [32]. The hostile dense stroma and the increased intra-tumoral pressure led to the hypo-vascularization of blood vessels surrounding tumours. Resultingly, this collapsed and dysfunctional blood matrix significantly prevents systemic chemotherapeutic agents from utilizing the Enhanced Permeability and Retention (EPR) effect for their passive diffusion into tumours [7,33,34]. Furthermore, recent studies have suggested that the hedgehog signalling pathway is responsible for the aggressive stromal compartment leading multiple studies to examine the effect of combining hedgehog inhibitors with different chemotherapeutic drugs [35,36]. Most importantly, almost 93% of PCs are overexpressed with multi-drug resistance (MDR-1) P-170 glycoprotein which poses another obstacle in the delivery of chemotherapeutic agents [37].

### 2.4. Current Diagnostic Strategies

The lack of early detection biomarkers and the deep anatomical position of the pancreas are major obstacles in the early diagnosis of pancreatic cancer. Although 70% of pancreatic ductal adenocarcinoma is present in the head of the pancreas (27) (Figure 1A), leading to obstructed bile duct and jaundice, PDAC is rarely diagnosed early. Moreover, most patients only develop mild symptoms in the advanced stages such as abdominal pain, weight loss, anorexia, nausea, and vomiting [38]. One of the major challenges in finding a biomarker for early detection, is that 80% of patients are diagnosed in late stages, hence, their blood samples only reflect the disease in the advanced stages [39]. Currently, Carbohydrate Antigen 19-9 (CA19-9) is the only FDA-approved biomarker for PDAC; however, it is neither specific nor sensitive in the early stages of cancer [40,41]. This proposed a new research direction for investigating extracellular vesicles (EVs) proteins such as Glypican 1 (GPC1) as a potential biomarker for the early stages of PC [42,43,44]. At this present stage, endoscopic ultrasound-guided fine-needle aspiration (EUS-FNA) is considered the first line diagnostic imaging procedure, as no other specific and standard imaging guideline is established [45]. 

Diagnosis and staging of the tumour are very important for choosing the appropriate treatment. Stages I and II are confined to the pancreas without the involvement of surrounding vessels, therefore, tumours are considered resectable at these stages. Stage III cancer is considered borderline resectable or locally advanced, as the tumours are yet to metastasize to the celiac axis or the mesenteric artery. Metastatic unresectable PC is presented in stage IV due to celiac axis or mesenteric artery infiltration [46]. 

Interestingly, according to the National Cancer Institute (NCI), early onset diabetes could be an early diagnostic alert for PC. It was reported that one out of every 100 people diagnosed with new-onset diabetes will also be diagnosed with PC within a time frame of 3 years. NCI has established a cohort study (Clinical Trials Identifier: NCT03731637) to study the incidence of pancreatic ductal adenocarcinoma including 10,000 patients who were diagnosed with new-onset diabetes [39,47]. With further understanding of the risk factors, continuous screening for higher-risk individuals is very crucial to diagnosing the cancer in its early stages.

### 2.5. Current Therapeutic Strategies and Limitations 

#### 2.5.1. Surgery

Although only 15–20% of pancreatic cancer patients are eligible for surgery by the time cancer is detected, resection remains the only curative option. Unfortunately, after resection, the recurrence rate is as high as 85%, due to the challenges associated with clear margins resection owing to the sensitive and vital structures surrounding the pancreas, such as the mesenteric artery, celiac axis, and blood vessels supplying the intestines [48]. However, surgical resection followed by adjuvant chemotherapy is the gold standard treatment for PC, as it increases the 5-year survival rate up to 30% [49]. For borderline resectable malignancies, neoadjuvant chemotherapy is encouraged to reduce tumour size; consequently, the tumour might then be qualified for resection.

Surgery is often performed when the cancer is confined to the head of the pancreas. The complex Whipple procedure is employed to remove the head, part of the small intestine, gallbladder, and bile duct. Other procedures include distal pancreatectomy and total pancreatectomy which involve resecting the tail of the pancreas or the entire pancreas, respectively [50]. 

#### 2.5.2. Chemotherapy

Gemcitabine (GEM) has been the first-line treatment for resectable and borderline resectable PC in the last two decades [51]. More recently, adjuvant and neoadjuvant FOLFIRINOX (a combination of 5-fluorouracil (5-FU), leucovorin, oxaliplatin, and irinotecan) is currently also referred to as the gold standard of care for PC [52,53]. Although this multimodal therapy offers a significantly higher overall survival rate compared to GEM monotherapy, its grade 3 and 4 toxic adverse effects significantly hinder its clinical applications, especially for the elderly and patients suffering from heart diseases [54]. The superiority of neoadjuvant chemotherapy over adjuvant remains a debate in ongoing clinical trials [55]. For patients unfit for FOLFIRINOX, a combination of GEM and capecitabine combination can be given [56]. In case the patients are not fit for combined chemotherapy, modified chemotherapy of GEM as the sole chemotherapeutics could be used [57]. For metastatic pancreatic adenocarcinoma, GEM and nab-Paclitaxel (PTX) are the first line of therapy to control tumour metastasizing, while the combination of nano liposomal irinotecan, 5-FU, and leucovorin are the second line of care [58]. In 2019, Olaparib was approved by FDA for patients with germline BRCA-mutated metastatic PC after receiving 16 weeks of chemotherapy [59].

#### 2.5.3. Radiotherapy

The role of radiotherapy in PC is controversial as clinical trials provide conflicting results [60]. In the clinic, chemoradiotherapy is currently being administered as neoadjuvant therapy for borderline resectable PC, to shrink tumour size for clearer margins resection [61]. In addition, it could be given as definitive and palliative therapy to relieve the pain of unresectable and metastatic pancreatic malignancies [62]. Radiotherapy is often administered in PC in form of External Beam Radiotherapy (EBRT) which includes multiple techniques with different types of radiation such as Stereotactic Body Radiation Therapy (SBRT), which will be discussed further in Section 3.4 [63].

#### 2.5.4. Immunotherapy

Although immunotherapy has been promising in various types of malignancies, its practice in the clinic for PC is very limited, due to the immunosuppressive microenvironment and the low mutated tumour nature of PC. Some studies suggested that the low count of dendric cells in the pancreatic tumour environment is the reason behind the low response to immunotherapy [53]. Although Pembrolizumab was approved by the FDA as an anti-programmed death-1 (PD-1) inhibitor for advanced PDAC, unfortunately, its effectiveness is limited to less than 1% of patients [64,65,66].

#### 2.5.5. Palliative Care and Pain Management

Unresectable PC is often presented with compilations such as gastric outlet obstruction (15–25%), jaundice, and extreme pain due to the tumour-infiltrated mesenteric or celiac plexus [67]. Currently, there is no standard palliative care for PC. The management regimen is constructed based on the patient’s prognosis, responsiveness to treatment, and efficacy-morbidity balance. Relieving duodenal or bile duct obstruction can be achieved through endoscopic stent placement, which is currently the most common procedure, in addition to bypass surgery, gastroenterostomy, or venting gastrostomy [68,69].

### 2.6. Recent Advances in Clinical Trials

#### 2.6.1. K-RAS Derived Therapies

More than 90% of PDAC patients expressed the mutations of the KRAS oncogene signalling pathway, which is known as the undruggable oncogene due to the difficulty in silencing it [70]. Sotorasib is currently the only FDA-approved K-RAS inhibitor, which covalently binds to GDP-bound KRAS^G12C^ mutated protein in lung cancer and no available drugs can directly target the mutated RAS gene [71]. Multiple clinical trials are studying different therapeutics that target different proteins associated with this gene (Table 1). Moreover, new studies have proven that by blocking the RAS pathway, cancer cells are forced to rely on autophagy, and when a combined drug is employed to block the autophagy, cancer cells may not be able to adapt, leading to their death [72].

#### 2.6.2. KRAS-LODER for PC

In addition to the challenging and hostile tumour microenvironment, the K-RAS mutation carries the heaviest burden in the progression of pancreatic precursor lesions into PDAC [73]. As a result, mastering the mechanisms of these two hallmarks can help us overcome the barriers in therapeutic approaches that leads to the immune evasion of pancreatic malignant cells. A novel biodegradable polymeric system was successfully fabricated by Silenseed, to locally deliver specific siRNA (siG12D-LODER) to pancreatic tumours. Potentially exhibiting antitumoral activity by inhibiting the translation of the KRAS proteins, this novel system was able to achieve up to 4 months of KRAS inhibition [74]. The system consists of intra-tumoral injectable poly (lactic-co-glycolic) acid (PLGA) based matrix, and it is currently undergoing stage II clinical trials (Table 1).

#### 2.6.3. Combination Immunotherapies

Recently, several clinical trials have been studying the effect of using a combination of immunotherapeutic agents for the treatment of PC, since monomodal immunotherapy has not been effective. Other trials are investigating the action of combining immunotherapy with other interventions, such as chemotherapy, radiotherapy, anti-angiogenic therapy, and stromal targeting agents (Table 1), for better treatment outcomes. Furthermore, multiple clinical trials are also evaluating the effect of natural killer cells and dendritic cells immunotherapies in the treatment of PCs [75].

**Table 1:** Current novel therapies in clinical trials. Information was obtained from clinicaltrials.gov.

Intervention	Delivered Drug(s)	Pancreatic Cancer Stage	Phase	Trial Identifier
**K-RAS-Targeting**	siG12D-LODER with chemotherapy	Locally Advanced PC	Phase II	NCT01676259
	Decitabine 50 MG	KRAS-dependant refractory metastatic/recurrent Pancreatic Adenocarcinoma	Phase II	NCT05360264
	Binimetinib Hydroxychloroquine	KRAS Mutant Metastatic PC	Phase I	NCT04132505
	Mesenchymal Stromal Cells-derived Exosomes with KRAS G12D siRNA	Metastatic Pancreas Cancer with KrasG12D Mutation	Phase I	NCT03608631
	mDC3/8-KRAS Vaccine	Pancreatic Ductal Adenocarcinoma	Phase I	NCT03592888
	Vemurafenib Sorafenib	Advanced KRAS G12D Mutated PC	Phase II	NCT05068752
	NALRINOX combination (modified FOLFIRINOX)	Resectable Pancreatic Ductal Adenocarcinoma	Phase II	NCT04010552
	ELI-002 2P	KRAS Mutated Pancreatic Ductal Adenocarcinoma	Phase I	NCT04853017
	Binimetinib Palbociclib	Operable KRAS-Positive Lung, Colorectal, or PC	Phase I	NCT04870034
**Immunotherapies Combinations**	Mitazalimab FOLFIRINOX	Metastatic Pancreatic Ductal Adenocarcinoma	Phase II	NCT04888312
	Cohort A: Nivolumab + Ipilimumab + Nab-paclitaxel + GEM Cohort B: Hydroxychloroquine + Ipilimumab + Nab-paclitaxel + GEM	Untreated Metastatic Pancreatic Adenocarcinoma	Phase I	NCT04787991
	Motixafortide, Cemiplimab, GEM, Nab-Paclitaxel	Pancreatic Adenocarcinoma	Phase II	NCT04543071
	Nivolumab + Irreversible Electroporation	Advanced Pancreatic Adenocarcinoma		NCT03080974
	Avelumab and Pepinemab	Metastatic Pancreatic Adenocarcinoma	Phase I and II	NCT05102721
	Cyclophosphamide GVAX PC Nivolumab Urelumab BMS-986253	Resectable Adenocarcinoma of the Pancreas	Phase II	NCT02451982
	Nivolumab FOLFIRINOX	Borderline Resectable PC	Early Phase I	NCT03970252
	Cyclophosphamide Nivolumab GVAX Pancreas Vaccine Stereotactic Body Radiation (SBRT)	Borderline Resectable PC	Phase II	NCT03161379
	APX005M Nivolumab Nab-Paclitaxel GEM	Metastatic Pancreatic Adenocarcinoma	Phase I and II	NCT03214250
	Anetumab Ravtansine GEM Hydrochloride Ipilimumab Nivolumab	Metastatic and recurrent Pancreatic Adenocarcinoma	Phase I and II	NCT03816358
	Irreversible Electroporation (IRE) Nivolumab Toll-Like Receptor 9	Metastatic Pancreatic Adenocarcinoma	Phase I	NCT04612530
	Nivolumab, ipilimumab Stereotactic body radiation therapy Low dose irradiation	Stage IV PC	Phase I	NCT05088889
	Pembrolizumab DEBIO1143	Adenocarcinoma of the Pancreas Adenocarcinoma of the Colon Adenocarcinoma of the Rectum	Phase I	NCT03871959
	Cyclophosphamide GVAX PC vaccine Pembrolizumab Stereotactic Body Radiation (SBRT)	Locally Advanced PC	Phase II	NCT02648282
	Stereotactic Body Radiation (SBRT) Nivolumab CCR2/CCR5 dual antagonist GVAX PC vaccine	Locally Advanced PC	Phase I and II	NCT03767582
	Anti-SEMA4D, Monoclonal Antibody VX15/2503, Ipilimumab, Nivolumab	Resectable Pancreatic and Colorectal Cancer	Phase I	NCT03373188
	Pembrolizumab Olaparib	Metastatic Pancreatic Adenocarcinoma	Phase II	NCT05093231
	Gene Mediated Cytotoxic Immunotherapy (GMCI™) (aglatimagene besadenovec + valacyclovir) + chemoradiation+ surgery	Advanced Non-Metastatic Pancreatic Adenocarcinoma	Phase II	NCT02446093
	Autologous Th-1 Dendritic Cell vaccine + chemotherapy	Pancreatic Adenocarcinoma	Phase I	NCT04157127

## 3. Locoregional Therapies

### 3.1. Localised Drug Delivery Systems (LDDs)

**Figure 2:**
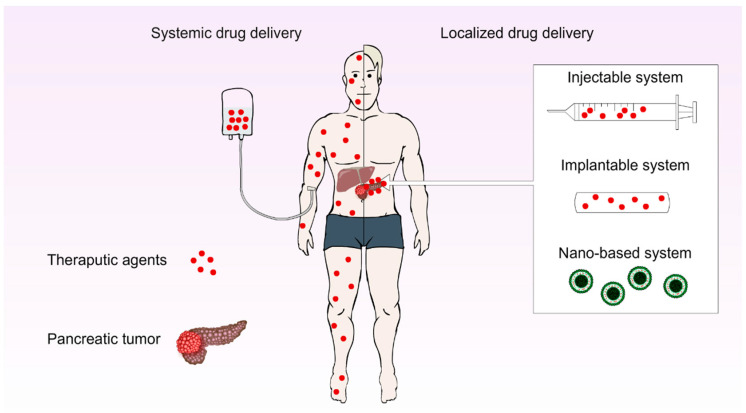
Illustration of localised vs. systemic therapy for the treatment of PC. Systemically injectables are designed to kill cancer cells that have migrated outside of the pancreas to organs elsewhere in the body. Localised therapy, including injectable, implantable, and micro/nano-based formulations, affect a specific area of the pancreas to control a wide margin of tumours. Image adapted from Aquid et al. [77].

The treatment options for PC in Section 2.5 above can be mainly classified into “locoregional” and “systemic” approaches, as demonstrated in Figure 2. Localised interventions, including surgery, various ablation therapies, external radiation therapy, intra-arterial infusion, and isolated upper perfusion, affect a specific area of the pancreas to minimize local tumour recurrence and reduce side effects on surrounding healthy tissues [76]. With this approach, wider margins around the tumour can be sufficiently damaged by chemo-agents, ensuring that the of risk local cancer re-growth will be minimised [77]. In contrast, systemic therapy involves the use of injectable formulations/devices to deliver drugs to the whole body, eliminating the potential tumours and potential metastasised tissues that have fled the pancreas to elsewhere in the body [77,78]. As mentioned earlier in Section 2.5.1., although surgery is favoured as being the most curative intervention for solid tumours, only 20% of patients are suitable for it based on disease staging [79]. In addition to this, achieving complete removals of tumour cells is challenging, and approximately 80% of these patients develop relapse [77,79]. As a result, additional post-surgery localised and systematic interventions are often employed, lessening the risk of tumour relapse and metastasis, and improving the overall chance of survival for patients [77].

One major hurdle in the treatment of PC is the predominantly elderly patient population, accounting for their poor overall health and low rate of successful treatment outcomes [77]. Therefore, post-surgery systematic therapy might not be ideal for the treatment and management of PC. This is due to the prolonged systemic contact with cytotoxic chemotherapeutics with higher concentrations, exposing the patient to a higher risk of systemic toxicities [80], adding burdens to the overall well-being of patients. Unlike systemic administration, when localised interventions are employed, cytotoxic agents are retained at a specific target site, reducing systemic drug exposure to minimal toxicity and maximizing treatment efficacy. As a result, there is a growing interest in a scientific movement toward finding novel localised interventions as alternatives for systemic therapy. One of the most prominent and promising approaches was the use of localised drug delivery systems (LDDSs). This was evident with the GLIADEL^®^ wafers—the very first FDA-approved carmustine—releasing implants for the treatment of newly diagnosed glioblastoma in 1997 [81,82]. A copolymer of 1,3-bis-(p-carboxyphenoxy) propane and sebacic acid in a 20:80 molar ratio was proven to be the best polymer matrix for the loading and release of carmustine, owing to its safety in primate brain [83] and the ability to protect the drug from hydrolytic degradation [84]. When used as an adjunct therapy to surgery and radiation, GLIADEL has proven its long-term effectiveness, significantly enhancing the overall survival outcome, without increasing toxicity [85,86]. Subsequently, in 2003, GLIADEL^®^ wafers were approved by FDA for the treatment of high-grade glioma (HGG)-grade III and grade IV [81]. As a result, GLIADEL^®^ wafer has been approved in 18 countries globally and was recognised as part of the treatment strategy for malignant gliomas by both the American Association of Neurological Surgeons/Congress of Neurological Surgeons (AANS/CNS) Joint Tumour Section and the National Comprehensive Cancer Network Clinical Practice Guidelines in Oncology [87,88].

The encouraging success of GLIADEL^®^ wafers has laid a strong foundation for more research towards developing localised delivery systems of chemotherapeutic agents into cancer after surgical resection, including pancreatic tumours to reduce the local recurrence. With the advent of numerous drug delivery systems (DDSs) or engineered “carriers” that introduce drugs into the body of the patients, the time-consuming drug development process of new alternative localised treatments has been sufficiently accelerated (41). The practice of drug delivery has been exploited extensively over the past decades for cancer therapy, resulting in numerous research studies on localised DDSs specifically for PC, with multiple levels of success. In the following section, recent advances in the synthesis of different localised DDSs for the treatment of pancreatic tumours in the period 2012–2022 will be discussed, with specific focus on injectable and implantable DDSs.

#### 3.1.1. Injectable DDSs

##### Gel-Based Systems

Amongst localised DDSs, injectable hydrogels were reported to be less invasive, with greater ease of administration compared to other systems implants [89,90,91]. Hydrogels are hydrophilic crosslinked 3D polymeric networks, possessing the capacity to swell and hold a significant amount of water [92]. The most feasible technology used for the synthesis of hydrogel is sol-gel, in which a colloidal solution (“sol”) undergoes hydrolysis and condensation reactions at a relatively low temperature to form 3D rigid networks (“gel”) [93]. Naturally occurring and synthetic hydrogels have been employed by many scientists to develop advanced localised DDSs, with the ability to shrink or expand in response to external stimulations such as temperature. Thermal-sensitive hydrogel possesses the ability to transport the incorporated drug to the specific site of action, enhancing the drug action through extended-release profile, reducing systemic exposure, and increasing the overall compliance of patients [94,95].

For instance, Phan et al. fabricated the temperature-sensitive nanohybrid hydrogels to deliver GEM to pancreatic tumours [96]. GEM was firstly introduced to interlayer galleries surfaces of montmorillonite (MMT) nanoparticles which act as cross-linkers, forming GEM-MMT/complexes. The resulting GEM-MTT/complexes were then incorporated into the synthetic thermal-sensitive poly(ε-caprolactone-*co*-lactide)-*b*-poly (ethylene glycol)-*b*-poly(ε-caprolactone-*co*-lactide) (PCLA–PEG–PCLA), generating the injectable GEM-loaded nanobiohybrid hydrogels. The final system could surpass the initial burst release of GEM, facilitating its extended-release profile. This effect was attributed to the ability of MTT nanoparticles to govern the diffusion rate of GEM out of the hydrogel matrix. GEM-loaded nanobiohybrid hydrogels also demonstrated good biocompatibility when exposed to 293T healthy kidney cells, with greater than 80% of cell viability even at the nanobiohybrid concentration of 2000 μg mL. GEM-loaded nanobiohybrid hydrogel exhibited a considerable level of tumour suppression in pancreatic tumour-bearing mice. Even though the incorporation of MMT remarkably enhanced the stability of the hydrogel, future studies should take into account the fact that MTT nanoparticles do suppress the overall biodegradability of the whole system.

**Figure 3:**
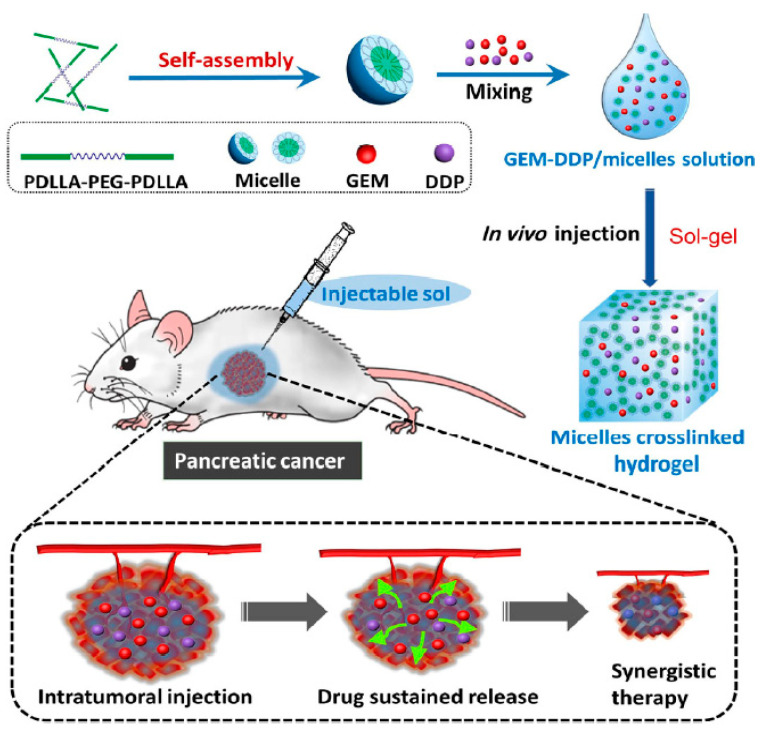
Schematic illustration demonstrating the mechanism of action of GEM/DDP-loaded thermal sensitive hydrogel. In water, the amphiphilic triblock copolymer (PDLLAPEG-PDLLA) underwent self-assembling into micelles at room temperature. GEM and DDP were dissolved into a predetermined amount of micelles solution to form a homogeneous (GEM-DDP/micelles) mixture. Upon exposure to body temperature, the resulting mixture spontaneously gelated into a cross-link hydrogel network (GEM-DDP/hydrogel). Following intra-tumoral injection, (GEM-DDP/hydrogel) dispersed around tumour sites, producing the sustained release of GEM and DPP, facilitating their synergic antitumour activity. Reprinted with permission from Shi et al. [91]. 2022, Springer Nature.

In a similar study, Shi and colleagues developed a thermo-sensitive hydrogel to co-deliver GEM and cis-platinum (DDP), to achieve the synergistic anticancer effects of these agents on pancreatic tumours. The biodegradable poly (D, L-lactide)-poly (ethylene glycol)-poly(D, L-lactide) (PDLLAPEG-PDLLA, PLEL) amphiphilic triblock copolymer was employed for the synthesis of the hydrogel. The GEM/DDP-loaded systems remained in the free flowable micellar liquid form at room temperature; upon intra-tumoral injection, the drug-loaded micelles dispersed around tumours and spontaneously assembled into physical crosslink hydrogel at body temperature, without any chemical reaction. The system achieved a long-term sustained release of both drugs, with 91.8% and 59.7% of drug released after 10 days, for GEM and DDP respectively. Furthermore, the dual-drug-loaded hydrogels demonstrated minimised systemic toxicity and superior anti-tumour efficacy on Bxpc-3 pancreatic cell lines and xenograft mouse model, compared to single-drug-loaded hydrogels [91] (Figure 3).

In another study conducted by Mao et al., liposomal paclitaxel (PTX) was incorporated with the thermosensitive poloxamer 407 and poloxamer 188 (P188), forming liposome gel (PTX-lip-gel) [97]. PTX-lip-gel had a mean particle size of 140.2 nm, demonstrating insignificant changes compared to native liposomal PTX (138.5 nm). Similarly, the zeta potential and morphology of the liposomal PTX remained relatively stable after being loaded into poloxamer gel matrix, suggesting that poloxamers did not interfere with the stability of the PTX liposomes. In addition to this, PTX-lip-gel demonstrated a more sustained and stable PTX release compared to liposomal PTX. Furthermore, PTX-lip-gel obtained a much larger intra-tumour PTX retention (41.61 ± 5.13%) than PTX-lip (3.7 ± 0.32%) 48 h post-intra-tumoral injection in S180 tumour-bearing mice. As a result, PTX-lip-gel significantly enhanced the tumour suppression effects and caused more extensive destruction to tumour tissues.

##### Nanoparticle (NP)-Based Systems

Nanotechnology has been extensively exploited by many research groups in the world to develop advanced DDSs for solid tumours. Nanocarriers possess various desirable characteristics, including nano-size, an abundance of surface chemical moieties, and the ability to host a large number of therapeutics cargos and protect them from systemic degradation, allowing a smaller quantity of incorporated therapeutics to be delivered to tumours over an extended period [98,99,100]. In recent years, NPs have been precisely engineered to bypass the obstacles associated with free drugs, promoting enhanced transportation of therapeutics across biological barriers [101]. One of the noble examples would be the use of hydrophilic nanocarriers to increase the concentration of hydrophobic drugs in target tissues at a predictable drug destiny in the body, by decreasing the probability of macrophage clearance following administration [102].

Albumin, an abundant hydrophilic component of the human blood, has been widely used in pharmaceutical formulation for this purpose, as evidenced by the success of the FDA-approved albumin-based nanomedicine Abraxane in the clinic [103]. In a study conducted by Noorani et al., albumin nanoparticles (ANPs)—synthesised by desolvation and thermal cross-linking methods—were used to transport the hydrophobic drug erlotinib to pancreatic tumours [104]. The resulting erlotinib-loaded-ANPs achieved an impressive nanosize of 9 nm, with drug loading (DL) and entrapment efficiency (EE) of 27% and 44%, respectively. Most importantly, a lower dose of erlotinib in ANPs was able to kill 50% of pancreatic adenocarcinoma ASPC-1 and PANC-1 cells, confirmed through MTT cytotoxicity assay. Even though the authors did not include the MTT assays on non-cancerous cell lines in this work, the findings suggested the enhanced anticancer effects of erlotinib-loaded-ANPs, while potentially reducing unwanted side effects of erlotinib.

Basel and colleagues reported the use of RAW264.7 cells, a type of monocyte-like tumour homing cells to transport magnetic iron oxide nanoparticles (ONPs) directly into the pancreatic tumour tissue, generating localised hyperthermia to kill tumours [105]. When injected intraperitoneally, RAW264.7 cells directly infiltrate tumour sites without infiltrating other organs, making them extremely promising carriers for localised targeting of tumours. Based on the MTT cytotoxicity assay, nanoparticles were loaded at 37.5 μg/mL iron to RAW264.7 cells to maximize the amount of iron-loaded, while preventing undesired toxicity. The temperature of ONPs-loaded-cells increased to 4.0 ± 0.7 °C after 15 min of alternating magnetic fields (AMF), which was greater than that of unloaded cells which were 1.0 ± 0.5 °C, observed under the same conditions. This confirmed the heat-generating effect of magnetic NPs. Resultingly, the system significantly increased the post-tumour injection survival outcome in mice bearing pancreatic tumours, with a 31% increase in average lifespan.

##### Material-Free DDSs

**Figure 4:**
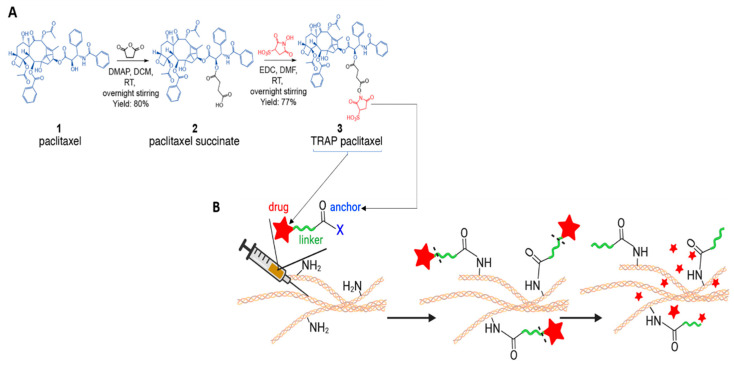
Graphical illustration of PTX-TRAP depots’ conceptual framework. (**A**) Synthesis scheme of TRAP PTX: PTX was dissolved in DCM and reacted with succinic anhydride in the presence of DMAP at room temperature to yield PTX succinic acid (PTX-SA). Purified PTX-SA was then reacted with EDC and sNHS in DMF at room temperature, yielding PTX-sNHS conjugate. (**B**) Following injection, PTX-sNHS conjugate reacted with the amines group on ECM, attaching PTX to the tumour tissues. After the linker moiety dissolved, PTX was released intratumorally. Reprinted and modified with permission from Pandit et al. [106]. 2022, Elsevier.

In addition to all the aforementioned studies in which the use of additional materials is needed for the drug carriers, there are a few studies in which localised drug delivery to pancreatic tumours can be achieved by altering the method of administration. In recently published research by Pandit et al., Tissue Reactive Anchoring Pharmaceuticals (TRAPs) technology was employed to enable the local drug depots at the pancreatic tumour sites [106]. In this study, purified paclitaxel succinic acid was reacted with 1-Ethyl-3-(3-dimethylaminopropyl) carbodiimide (EDC) and 1-Hydroxy-2,5-dioxopyrrolidine-3-sulfonic acid (sNHS), forming paclitaxel sNHS conjugated (TRAP-PTX) (Figure 4A). Following injection, TRAP-PTX diffused throughout the tumour and reacted with the amines in the tissue extracellular matrix (ECM), anchoring PTX to the tissue ECM via amide bonds (Figure 4B). Intra-tumoral injection of TRAP-PTX demonstrated a controlled release profile of PTX and maintained its concentrations in the tumour ECM for an extended period of time. Furthermore, TRAP-PTX possessed significantly higher apoptosis and viability of tumours in KPC 4662 pancreatic adenocarcinoma mouse model, compared to the unanchored PTX. As a non-viscous carrier, TRAP products induced the radial penetration of small therapeutic molecules in the dense stroma, making them very promising vehicles for pancreatic tumour drug delivery.

It is also possible to improve the localised drug delivery to tumours through the manipulation of injectable devices. Ohara et al. has improved the Endoscopic Ultrasound-guided Fine Needle Injection (EUS-FNI) technique, with a newly designed ‘Multiple Injectable Needle’ (MIN), to deliver drugs to refractory pancreatic tumours [107]. MIN consists of a manipulatable 25-gauge inner needle embedded in a 19-gauge rotatable outer needle, capable of injecting a potential drug into the tumour in multiple directions at a puncture site. Ethanol was used as a model drug and EUS was used to observe its injection into pancreatic orthotopic tumours. MIN demonstrated its ability to enhance the distribution of ethanol compared to straight-type needles, suggesting its great potential in EUS-FNI as a novel tool for PC treatment.

Recent innovations of mentioned injectable DDSs are summarised in Table 2, with respect to their compositions, method of production, and release profile of incorporated drugs.

#### 3.1.2. Implantable DDSs

The very first implantable DDSs in modern medicines were conceptualised by Deansby and Parkes in 1938. In this study, compressed pellets of crystalline estrone were subcutaneously (SC) implanted in castrated male chickens [109] to study their effects. From these early discoveries, implantable DDSs have proven themselves as revolutionised, personalised, and precision tools in many fields, including oncology. With many advantages and desirable features, implantable DDSs enable targeted and localised drug delivery to achieve therapeutic effects with lower drug concentrations, hence minimizing potential side effects and increasing patient compliance [110]. As mentioned earlier in this review, GLIADEL^®^ wafers embody all desirable characteristics of implantable DDSs, building an excellent foundation for more research towards many more advanced systems, including those intended for the treatment of pancreatic tumours. These systems will be discussed in the following section.

##### Casted Implants

Casting is the process in which a melted or dissolved polymer solution is poured into a cast and subsequently solidifies into implants with chemical or physical recrystallization [111]. Owing to flexibility in the choice of polymers, including thermoplastic biopolymers and natural/synthetic hydrogels, casting is considered one of the most well-known techniques used to fabricate implantable DDSs [111]. For example, GEM-incorporated polyurethane (GIPU) films were developed to deliver GEM at a various concentration to pancreatic tumours. The prolonged delivery rate of GEM was observed, with obtained cumulative absorbance after 16 days [112]. Similarly, an implantable GEM-containing membrane was also fabricated using the casting method, for the treatment of inoperable malignant biliary obstructions [113]. GEM-containing membrane derived from this study demonstrated the ability to promote the Casitas B-lineage lymphoma (c-CBL)-mediated degradation of epidermal growth factor receptor (EGFR) in xenograft tumours, inducing tumour death through the inhibition of their proliferation, angiogenesis, and epithelial-mesenchymal transition.

##### Dip Coated Implants

Dip coating is the most well-known liquid-phase deposition application of sol-gel technique, used to fabricate thin film coating in biological research [93,114]. This process involved immersing a substrate into a solution at a designated rate; following the evaporation of the liquid component, the substrate is left with the coated solid thin film [115,116]. Therefore, the adhesion and cohesive forces between the substrate and the dipping solution play an important role in the success of the coating process [117]. Several studies have employed the use of dip coating in the formation of implantable DDSs for PC treatment. For example, Byrne and colleagues developed dip-coated iontophoretic devices to deliver FOLFIRINOX to orthotopic pancreatic tumours [118]. In this study, a platinum disk soldered to a stainless-steel cable wire was dipped in a polyurethane reservoir at 60 °C, allowing the polymer to coat this steel wire. The resulting by-product was then threaded through multi-luminal tubing, and its interface was encased with extra polymer in heat-shrink tubing. Finally, a semipermeable 14K cellulose membrane adhered to the device for an enclosure. The resulting devices achieved almost an order of magnitude increase in tumour exposure in the orthotopic PDX model of PC, with substantially lower plasma concentrations, outperforming the control group of I.V administration and saline-containing devices. Furthermore, the delivery of FOLFIRNOX via iontophoretic devices demonstrated no obvious evidence of tissue toxicity, with or equivalent treatment tolerance on mice as compared with I.V FOLFIRINOX. In a follow-up study, Byrne and colleagues explained the effect of formulation on the electrotransport of the drugs in the FOLFIRINOX combination, to optimize delivery efficiency [119]. It was concluded that when iontophoretic FOLFIRINOX was divided into two solutions, higher levels of cytotoxic drugs in pancreatic tumours can be obtained, compared to drugs combined in one single solution or delivered individually.

In another study conducted by Indolfi et al., an implantable poly(lactic-co-glycolic)-coated stainless-steel device was developed to provide local delivery of PTX to pancreatic tumours [120]. By tuning drug content and polymer concentration, the authors successfully achieved linear and prolonged PTX release of up to 60 days. This delay of drug release through the modification of polymer concentration is an excellent feature of this research, as it allows the surgical wounds sufficient time to heal before chemotherapy begins. In addition, this local delivery device achieved a 12-fold and 2-fold reduction of viable tumour volume, in PDAC-3 and PDAC-6 tumour-bearing mice, respectively; and better control of local tumour dissemination compared to those given intravenous PTX. Overall, the findings of this study offer promising potential not only for improving the survival outcome but also for enhancing the quality of life for cancer sufferers. However, with these two mentioned studies [118,120], non-biodegradable, non-absorbable materials were used. Therefore, it would require a further surgical procedure for device removal increasing the risk of infection and reducing patient’s inconvenience.

##### Electrospun Fibrous Implants

The electrospinning technique is used to fabricate fibres from various natural and synthetic polymers, obtaining fibrous materials with few nanometres to micron-size diameters [121,122]. Electrospinning is the conversion of a polymeric solution into solid nanofibers under electrical force [121]. Electrospun materials have opened a new horizon to drug delivery, owing to their high loading capacity, tuneable designs by controlling viscosity, molecular weight, humidity of the electrospinning chamber, and the retention of the bioactivity of therapeutics and biomolecules despite the high voltage used for electrospinning [122,123,124].

**Figure 5:**
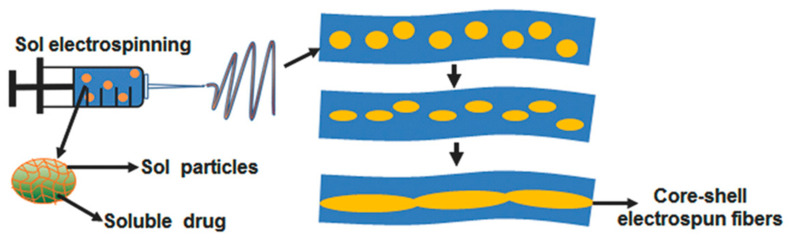
Schematic illustration of core-shell electrospun fibres containing GEM. GEM-HA-sol was firstly dispersed into PLA solution to form an electrospinning solution. Under the application of electrospinning technique, GEM-containing core-shell electrospun fibres are formed, namely GEM@PLA-HA. Reprinted with permission from Xia et al. [125]. 2022, John Wiley and Sons.

A core-shell electrospun fibre system comprising poly(l-lactide) (PLA) and hyaluronan (HA) was fabricated by Xia et al. for the treatment and prevention of pancreatic tumour recurrence [125]. In this system, GEM was firstly dissolved in HA hydrosol, forming GEM-HA-sol which was then dispersed into PLA solution to emulsify into an electrospinning solution. The electrospinning technique was then applied to form the core-shell fibres containing GEM (Figure 5). GEM@PLA-HA electrospun membranes were easily implanted onto the surface of tumours and exhibited antitumour effects. However, these effects were not superior to the treatment with single GEM in animal-bearing integrated xenograft pancreatic tumour. The authors attributed this observation to the fibrous tissue around the tumour which acted as a barrier to GEM penetration. Despite this, the GEM@PLA-HA electrospun membrane was more efficient in inhibiting the growth of residual tumours, and significantly reduced liver toxicity, compared to GEM given intravenously.

Similarly, in a recently published study, electrospinning was employed for the fabrication of poly(L-lactic acid) (PLLA) non-woven sheets to deliver GEM to pancreatic tumours [126]. A long-term sustained release pattern was observed, with cumulative in vitro GEM release of approximately 35% and 36% after 30 and 60 days, respectively, whereas that observed in vivo was 61% and 68%, respectively. The authors attributed the sustained release profile of the system to the crystallization of PLLA through which GEM was homogeneously dispersed, and subsequently crystallised by field spinning during fibre formation. When implanted close to the tumour site, the inhibition of tumour growth in the system lasted approximately 28 days.

In addition to monotherapy, several implantable electrospun fibres were developed to deliver a combination of therapeutics to tumours. For example, Zhan et al. fabricated an electrospun scaffold from polyglycolide-co-trimethylene carbonate (PGA-TMC) and gelatin for the localised delivery of FOLFIRINOX [127]. It was reported that a lower dosage of FOLFIRINOX delivered through this scaffold possessed comparable antitumorigenic and antimetastatic effects, roughly two-thirds of traditional regional chemotherapy. Owing to the ability to destroy CD133+CXCR4+ cells in tumour tissues, FOLFIRINOX eluting electrospun scaffolds successfully stabilised tumorigenesis and prevented hepatic metastasis.

Additionally, to deliver a combination of GEM and PTX, Wade and colleagues employed two biodegradable polymers, alginate (Alg) and polycaprolactone (PCL), for the synthesis of wet-spun fibres [128]. GEM was firstly loaded into the water-soluble Alg core while PTX was loaded into the PCL shell. The systems achieved an EE of 48.5% for GEM, and 89.3% for PTX. They also demonstrated a significant reduction in cell viability on PDAC exocrine pancreas tumour cell line. The in vivo data obtained from the xenograft PDAC mouse model revealed a significant reduction in tumour volume of (GEM+PTX) implant, equivalent to that of intratumorally (GEM+PTX). Most importantly, this effect is superior to the I.V. administration of the two drugs. The (GEM+PTX) implant derived from this study also demonstrated an excellent safety profile, with no observed adverse side effects, making it a promising treatment option for PDAC patients in the future.

Recent innovations of mentioned injectable DDSs are summarised in Table 3, concerning their compositions, method of production, and release profile of incorporated drugs.

### 3.2. Thermal/Energy Ablation Therapy

As mentioned before, PC is usually detected in the late stages and is more common in elderly patients where surgical resection is unfeasible. Therefore, thermal ablation offers safer and easier alternative treatment options with lower mortality, lower cost, and potential immunomodulation [132,133]. Thermal ablation is associated with multiple disadvantages such as incomplete necrosis causing tumour recurrence and the variation in the clinical outcomes between patients, in addition to the difficulty of applying thermal ablation in tumours near major blood vessels due to the heat sink effect especially radiofrequency ablation (RFA) and with less extent microwave ablation (MW) [134,135]. In this section different thermal ablation therapies will be discussed in relation to pancreatic tumours.

#### 3.2.1. Radiofrequency Ablation (RFA)

Although radiofrequency ablation is not used as a sole intervention in cancer treatment, its effect in treating and relieving pain in many solid tumours is well established [136,137]. In addition to this, it is widely used in relieving long-term pain in the neck and back and treatment of chronic venous insufficiency [138]. On the other hand, the role of RFA in the treatment of pancreatic tumours is still under investigation through multiple clinical trials (Table 4). The technique utilizes an electrode to deliver high and focused thermal energy (60–100 °C) through high alternating radio waves, causing tumours to shrink due to protein denaturation and coagulative necrosis [133]. Recent studies have supported the ability of RFA to stimulate the antitumour immunity, through releasing various extracellular immunogenic substrates, such as heat shock proteins, in addition to increased levels of proinflammatory cytokines [133,139]. Unfortunately, EUS-RFA is associated with several adverse effects including gastrointestinal haemorrhage, sepsis, wound infection, and acute pancreatitis depending on the radiofrequency power [140,141]. Although it has not been studied in PC yet, multiple studies have proved that insufficient and incomplete radiofrequency ablation can promote the progression of hepatocellular carcinoma metastasis [142,143].

RFA therapy could be applied to the pancreatic tumour through multiple techniques, either by using an endoscopic ultrasound guide (EUS) through stomach/duodenum, CT scan, or ultrasound image of the abdomen, aiding the surgeons in achieving minimal damage to the surrounding organs. RFA could also be employed intraoperatively, especially for patients with bile duct obstruction or for tumours deemed unresectable in the operating room [145]. In a recent systematic review with pool analysis conducted by Spadaccin et al., 120 patients with pancreatic malignancies underwent endoscopic ultrasound-guided radiofrequency ablation (EUS-RFA). The pooled obtained a success rate of 99%, with an overall adverse effects rate of 8%, without any mortality related to the procedure [146]. In another observational study, 14 patients with locally advanced unresectable PC and another 8 with metastatic PC undertook chemotherapy after EUS-RFA. An overall survival duration of 24 months was reported, with roughly 4% procedure-related adverse effects, including peritonitis and abdominal pains. This study suggested the promising outcomes of combining EUS-RFA with chemotherapy [147]. Indeed, after patients undergoing EUS-RFA, currently available imaging techniques are not capable of differentiating between necrotic tissues and tumour cells [148]. Mazzawi et al. presented a case report of the advantageous role of EUS elastography in staging the status of the pancreatic tumour subsequent RFA through grading the hardness of a lesion [149]. In another study conducted by Yan et al., an overall survival rate of 10.77 months was obtained in 74 patients undergoing RFA with subsequent chemotherapy, compared to 5.77 months of those receiving chemotherapy alone [150].

Interestingly, a randomised controlled trial revealed a conflicting conclusion that radiofrequency ablation alone showed the lack of superiority over traditional chemotherapy, and it should only be considered a part of multimodal therapies for localised advanced PC [151]. To investigate the effect of RFA and chemotherapy against standard chemotherapy alone in PC, a current multicentre randomised clinical trial is being conducted [152]. However, to our knowledge, no randomised clinical trial comparing the outcome of RFA and surgical resection in PC has been conducted.

#### 3.2.2. Microwave Ablation (MWA)

MWA is a relatively newer thermal technique that utilizes one or more microwave antennas inserted directly into tumours, especially liver, kidney, and bone malignancies [153]. Similar to RFA, microwave ablation causes tumour necrosis through hyperthermia utilizing electromagnetic waves [154]. In contradiction to RFA, microwave ablation is capable of producing higher temperatures in shorter durations; therefore, the technique produces a wider and more predictable ablation radius owing to its independence from the conduction through tissues, making the technique more suitable for larger tumours [133,155]. Alternatively, RFA shows significantly higher necrosis and immune response compared to microwave ablation, in addition to lower recurrence. Through the findings of recent comparative studies between microwave and radiofrequency ablation in liver malignancies, no significant difference in the clinical outcomes between both techniques was obtained, despite microwave being superior in larger malignancies [155,156,157].

Although microwave ablation is a well-established intervention in hepatic malignancies, studies regarding implementing the technique in PC are very limited. The most recent one was conducted in 2017 by Vogl and colleagues, with 100% efficacy with minor adverse effects, and 10% local progression reported in 20 patients who underwent microwave ablation for locally advanced PC [158]. Clinical trials investigating the effect of microwave ablation, along with different interventions, are presented in Table 4.

#### 3.2.3. High Intensity Focused Ultrasound Ablation (HIFU)

Similar to RFA, HIFU is a hyperthermic technique in which ultrasound beams are used to generate acoustic energy to elevate tumour temperature (>60 °C). Unlike RFA and microwave ablation, HIFU is considered non-invasive, with no required anaesthesia [159] and minimal effect on the surrounding tissues [133,160]. The technique causes coagulative necrosis to the tumour tissue and acoustic cavitation, leading to the collapse of cancer cells [161]. HIFU is usually indicated for patients with unresectable PC or unfit for surgeries, unresponsive to chemotherapy, and as a palliative treatment. Fergadi et al. conducted a meta-analysis study involving 939 patients with PC, revealing that HIFU when paired with subsequent chemotherapy is a safe intervention, inducing an overall survival rate and less discomfort compared to chemotherapy alone [162]. Similarly, in another study conducted on 176 patients with stage III and IV PC receiving HIFU paired with traditional chemotherapy, an increased survival time and more than 60% pain relief were obtained [163]. Zhao et al. investigated the effects of patients receiving two types of HIFU, traditional or low-power cumulative HIFU, and the former offered significantly higher pain response and overall survival [164]. An interesting case report demonstrated a promising result of a 61-year-old male patient who underwent focused ultrasound ablation, the updated scans post-treatment demonstrated a 12-month progression-free [165].

A large body of scientific precedence has demonstrated the significant benefit of HIFU in improving the quality of life for patients, with enhanced tumour control and reduced discomfort [166,167]. As a result, multiple clinical trials are being conducted, to investigate further the effect of HIFU on PC (Table 4).

#### 3.2.4. Cryoablation

Cryoablation is one of the oldest ablation techniques, the use of which started in the 1980s. Since then, it has been used in the treatment of skin disorders and different types of cancer [168]. Cryoablation is usually applied percutaneously or intraoperatively. It can be combined with different modalities including radiotherapy, chemotherapy, and immunotherapy. This technique utilizes liquified gas such as argon gas to decrease the temperature (−140 to − 150°C) by the effect of gas expansion causing vascular and cellular injuries [133]. In addition, the technique causes modulation of the immune system with much higher immunogenicity and immunosuppressive effect in comparison to other ablation modalities [133,169]. Moreover, another main advantage of cryoablation is the visible “ice ball” which is visible and controlled through imaging, reducing the damage to surrounding tissues [168]. On the other hand, studies have presented that cryoablation effect needs to extend 1 cm beyond tumour margins to ensure total ablation [170]. The main complications of the procedure are acute pancreatitis, pancreatic or gallbladder fistula, intrabdominal bleeding, and jaundice [171]. Liquid nitrogen is considered more convenient than argon gas, owing to its ability to offer faster freezing with lower temperature and shorter ablation time, together with the ability to use in normal operating room settings [172]. In a recently published study, when 10 patients with locally advanced PC underwent cryoablation and ultrasonography guide, a 70% success rate was obtained, with complete ablation, minor adverse effects, and significantly lower pain scores [173].

Li et al. compared the pain management effect of cryoablation with irreversible electroporation and reported no significant difference between the two techniques for managing pain [174]. Niu et al. compared the effect of cryoablation paired with subsequent immunotherapy against mono-administration of immunotherapy, chemotherapy, and cryoablation in patients with PC [175]. Cryoimmunotherapy showed significantly higher overall survival compared to other groups. In other research, Song et al. conducted a retrospective study of 118 patients with advanced PC, receiving either bypass surgery with cryoablation or bypass surgery alone. No significant difference between the two groups in terms of prognosis and pain relief was obtained, despite the reduction in tumour size with the use of cryoablation [176].

The in vitro effect of cryoablation has been studied in multiple articles. For example, Baust et al. subjected PDAC-1 cell lines to cryoablation, chemotherapy of GEM/or oxaliplatin, and a combination of these two modalities [177]. The findings from this study suggested that a temperature of −15 °C showed partial cell death, while complete cell death could be achieved at −25 °C only. However, in case the combined chemotherapy was used, enhanced cell death was achieved at −15 °C, suggesting the potential therapeutic effect of combining cryoablation with chemotherapy. In another study conducted by Baumann and colleagues, the response of different pancreatic cell lines (PANC-1 and BxPC-3) to different temperatures was investigated. At −10 and −15 °C, minimal effect on cell viability was obtained, while complete cell death was achieved at −25 °C and 50 °C [178]. Clinical data on cryoablation in PC are limited and MRCT is required to further study the technique. As a result, only one clinical trial studied the effect of cryoablation in PC as per Table 4.

### 3.3. Irreversible Electroporation (IRE)

IRE is an electrical ablative technique exploiting direct high voltage (up to 3 kV) to induce nanopores in the cancer cell membrane, causing high permeability and subsequent cell death [179]. NanoKnife^®^ System is the device usually employed in delivering the electrical current through multiple probes, with a minimum of two monopolar probes to be inserted, creating the ablation zone. IRE can be utilised percutaneously or intra-operatively with anaesthesia [180]. Thomson et al. were the first to investigate the safety of IRE in humans; since then, IRE has gained extensive attention in the field of cancer ablation [181]. IRE is currently used as a cytoreductive intervention for patients with locally advanced PC; hence, more research is required to utilize IRE in borderline resectable PC [182].

IRE has many benefits over thermal ablation techniques. For example, due to the lack of heat sink effect in IRE, the technique can be used in tumours surrounding sensitive structures, such as nearby blood vessels or connective tissues [183]. Another advantage of IRE in PC is its immunomodulation and immunosuppression effect, by increasing T-cells infiltration and stimulating macrophages [184,185,186]. A retrospective study compared two groups of LAPC patients who received either IRE with toripalimab or IRE alone, in which the former showed significantly higher overall and progression-free survival [187]. To further study the effect of IRE on pancreatic tumour’s immune response, 62 patients in a randomised study were given either IRE or combined with single or multiple allogeneic γδ T cells infusion [188]. According to the obtained results, significantly higher overall and progression-free survival was achieved in the combined therapy group; multiple infusions demonstrated superior efficacy over single infusion treatment. He et al. conducted a comparative study about the long-term survival of patients with locally advanced PC, between patients who underwent IRE after neoadjuvant chemotherapy and those receiving the c standard treatment (neoadjuvant and R0 resection) or chemotherapy alone. The study found that neoadjuvant chemotherapy followed by resection or IRE showed similar outcomes to the standard treatment [189]. In another study where 40 patients with stage III LAPC were treated with intraoperative IRE and neoadjuvant chemotherapy, 8% of the patients achieved 36 months of overall survival [190]. Several other studies have also demonstrated the enhanced efficacy when IRE was used in combination with other therapeutics [191,192,193]. Unfortunately, severe abdominal pain due to severe duodenal inflammation with wall thickening could result from IRE treatment [194].

To study the effect of IRE on patients’ quality of life and pain level, Flak et al. examined the data of non-metastatic patients who underwent IRE from 2013 to 2019, using linear mixed models. Unfortunately, no positive effect was shown on the quality of life and pain levels; hence, the authors suggested that IRE should not be considered a palliative treatment [195].

To optimize the patient criteria for IRE, a recent multi-institutional study concluded that patients with normal *Carbohydrate Antigen 19-9* levels before surgery and avoiding radiation therapy before IRE, in addition to administering FOLFIRINOX plus GEM or abraxane induction chemotherapy, are good interpreters of better survival rate after IRE [196]. Moreover, IRE is contraindicated with cardiac arrhythmia pacemakers [179]. Multiple clinical trials are currently studying the effect of IRE alone or with different interventions (Table 3).

### 3.4. Stereotactic Body Radiotherapy (SBRT) or Stereotactic Ablative Radiotherapy (SABR)

SBRT is a type of radiotherapy which utilizes focused beams to shrink tumours and blood vessels, with minimal effect on surrounding tissues. SBRT can be applied to different parts of the body including the lung, spine, neck, and liver, moreover, the technique is currently being directed into pancreatic tumours. Two technologies currently being employed are *Linear Accelerator* (LINAC) which exploits photons through a fractioned x-ray beam, and another more recent technology using proton beams in charged particle radiosurgery [197]. As SBRT is considered a non-invasive treatment and requires no surgical incision, patients experience less severe adverse effects compared to conventional radiotherapy [198]. SBRT is usually used after surgery to ensure R0 resection and kill the remaining cancerous cells; in addition, the technique can be utilised as palliative treatment in case LAPC has yet to metastasize [199].

Although SBRT presented the highest response rate, the overall and progression-free survival were found to be comparable to *Three-Dimensional Conformal Radiation Therapy* (3DCRT) and *Intensity Modulated Radiation Therapy* (IMRT) [200]. Similarly, the overall survival and toxicity in patients receiving SBRT were similar to that of *Conventional Fractionated Chemoradiation* (CRT) or chemotherapy alone [201,202]. In the preclinical setting, SBRT was reported to induce immunogenic tumour death in PDAC [203]. To further support these findings, Mills et al. collected samples of patients with resectable PDAC who underwent SBRT a week before surgical resection, and the analysis showed a higher immune-related cell death and lower malignant cell density, compared to samples of patients who did not undergo any interventions before surgery [204].

Shen et al. studied the efficacy of re-irradiation with SBRT in 24 patients with locally recurrent PC after an initial SBRT. Median overall survival from the first SBRT course was 26 months, and 14 months for the second course. The overall response rate and disease control rate were 50% and 13%, and 100% and 86.9% after each course of SBRT, respectively. This study highlighted the use of SBRT as a promising therapeutic option for recurrent PC, causing minimal toxicity and achieving good pain relief [205].

An open label, randomised, controlled phase II clinical trial studied the therapeutic effect of combining SBRT with immunotherapy pembrolizumab and trametinib against SBRT combined with GEM in 170 patients in total with recurrent PC. Results showed a higher median overall survival rate in SBRT with pembrolizumab and trametinib; in addition, severe adverse effects were more dominant in SBRT and pembrolizumab and trametinib (22%) compared to 14% in SBRT plus GEM [206]. A phase III trial is required to further study these findings. To further study the effect of combining immunotherapy with SBRT, a phase II clinical trial compared the therapeutic effect of SBRT+ nivolumab with or without ipilimumab in 84 patients with refractory metastatic PC. Although combining nivolumab with ipilimumab + SBRT enhanced the clinical benefits rate and increased the number of patients with partial response (median duration 5.4 months), the additive effect of SBRT in this trial was unknown (ClinicalTrials.gov identifier: NCT02866383) [207]. Teriaca et al. reported the long-term effects of SBRT in a multicentre phase II trial studying the effect of treating patients with LAPC 8 cycles of FOLFIRINOX followed by SBRT; after SBRT, few patients underwent radical resection. In the long-term results, the SBRT group presented a significantly higher overall survival of 18 months compared to 5 months in the non-SBRT group. Moreover, patients who underwent radical surgery had 3-years overall survival of 40% against 6.5% in a non-surgical group [208]. This clinical trial presented SBRT as neoadjuvant therapy to increase the probability of radical resection for patients with LAPC. Multiple clinical trials are currently recruiting to further asses the role of SBRT combined with various therapeutic interventions for different stages (Table 4).

### 3.5. Intra-Arterial Infusion Chemotherapy or Transcatheter Arterial Infusion

Intra-arterial infusion (IAI) is a therapeutic intervention that involves delivering chemotherapeutic agents directly to tumour site through catheter placement. Compared to systemic chemotherapy, IAI has the potential to increase local chemotherapeutic concentration and decrease systemic toxicities [209]. IAI has been successfully used in many solid tumours, specifically in primary and metastatic liver tumours. The efficacy of the technique in treating PC has yet to be fully established, due to the difficulty in applying the catheter in the complicated vascular structure surrounding the pancreas [210]. Furthermore, the double catheter placement in the main arteries supplying the pancreas, the celiac artery, and the superior mesenteric artery (SMA) is a highly complicated process.

In an attempt to unify the arteries supplying the pancreas and to simplify the technique, new studies have implemented embolization (blocking blood vessels using coils) of SMA and its all branches [211]. Due to the aggressive nature of cancer and the late diagnosis in the advanced and metastatic stages where the tumour developed new vasculature, the unification of the blood supply might be difficult for some patients [212]. RenovoCath^®^: a product fabricated based on this concept was granted a new 510(k) Clearance by the FDA in 2021. This system has been developed to deliver GEM through a dual-balloon infusion catheter that is inserted into the artery adjacent to tumour, pushing GEM through the wall with minimal side effects for the surrounding tissues [213]. The system is currently recruiting for phase III TIGeR-PaC clinical trial [214]. Moreover, multiple clinical trials are currently studying the safety and efficacy of intra-arterial infusion of chemotherapy against I.V administration (Table 4).

Using RenovoCath™ as a model, Rosemurgy et al. studied the safety and efficacy of intra-arterial infusion of GEM in 20 patients with LAPC [215]. Normal liver and pancreatic enzymes were reported. In addition, 73.3% of patients demonstrated disease stability while 20% displaced tumour progression. These results suggested the excellent potential of the RenovoCath system for the treatment of patients with LAPC. In a recent case report, Ranieri et al. treated a patient with LAPC using an intra-arterial infusion of FOLFIRINOX [211]. Three chemotherapeutic cycles were administered with no side effects. Despite the impressive tumour response, the patient passed away due to a lung infection at the end of the third cycle. Other patients are currently being enrolled to further confirm these results.

In a retrospective study, 115 patients with locally advanced or metastatic PC ineligible for systemic chemotherapy underwent IAI between 2007 and 2017 [209]. Disease control was achieved in 62.6% of patients, with median overall survival of 147 days. In addition, results revealed a significant increase in disease control and progression-free and overall survival in patients who underwent more than one ITI session, without major complications. In another conducted on 354 patients, 20% of which suffered from metastatic PC, 22.5% received two or more cycles of intra-arterial infusion of GEM, while the remaining completed only one cycle [216]. It was revealed that receiving two or more cycles of IAI did not improve the median overall survival significantly (from 6 months to 7 months).

Since the most common PC metastasis is in liver tumours, multiple articles studied the effect of infusing chemotherapy on both pancreas and the liver. For example, Sasada et al. compared the efficacy of intra-arterial infusion of 5-FU and cisplatin in the pancreas and liver in 16 patients with locally advanced and metastatic pancreatic cancer with the historical data for patients who underwent other modalities. Patients with locally advanced pancreatic cancer presented significantly higher overall survival compared to control. However, patients with liver metastasis showed no favourable results, suggesting that the technique is only effective in locally advanced PC with no distant metastasis [217].

In an attempt to investigate the clinical outcome of combining IAI with other interventions, Timmer et al. evaluated the efficacy and safety of endovascular seed implant of iodine-125 (125I) and stent placement in patients with LAPC receiving a regional intra-arterial infusion of chemotherapy. This treatment regimen demonstrated a 100% success rate, a median survival rate of 10.7 months, and 83% disease control [218]. As mentioned in Section 2.4., Glypican 1 (GPC1) is being investigated as an extracellular vesicular biomarker for the early detection of PDAC. Therefore, Qian et al. studied the possibility of using GPC1 as a prognostic biomarker for patients receiving intra-arterial chemotherapy. Resultingly, the levels of GPC1 and extracellular vesicles (EVs) were significantly higher in PDAC patients, compared to healthy individuals. In addition to this, the levels of GPCI and EVs decreased significantly while the overall survival increased post IAI treatment [219].

### 3.6. Isolated Upper Abdominal Perfusion

Isolated perfusion is a well-established technique used in delivering therapeutic drugs locally to reduce their systemic toxicity [220]. Depending on tumour site, there are different types of regional perfusion therapies that can be employed, such as *Hepatic Arterial Perfusion*, *Isolated Limb Perfusion,* and *Hyperthermic Intraperitoneal Chemoperfusion* (HIPEC) [221]. The research on the efficacy of these interventions in PC is still in its infancy, with very few clinical studies demonstrating promising results.

Delivering chemotherapies through upper abdominal perfusion for PC is achieved in two steps. The 1st step involves inserting a stop-flow balloon catheter through the aorta and inflating beneath the celiac trunk, followed by the 2nd step which is isolated hypoxic upper perfusion with high chemotherapeutic concentration [222]. To compare the clinical outcome of isolated upper abdominal perfusion and intra-arterial infusion, a retrospective cohort study including 454 patients with stage III and VI advanced PC was conducted [222]. In this study, a combination of cisplatin, adriamycin, and mitomycin was infused through a celiac axis catheter in 233 patients; in contrast, 221 patients underwent upped abdominal perfusion with the same drug combination. The upper abdominal perfusion showed significantly greater median survival rates in stage III participants. However, in stage IV patients, abdominal perfusion showed a slight improvement in median survival. To confirm these preliminary results and to further study the effect of upper abdominal perfusion on the quality of life of pancreatic patients, the same research group continued a study involving another 221 patients [223]. Almost half of Stage III patients achieved one-year survival, and 21.7% achieved 3-year survival. While in stage IV, one-year and three-year survivals were 37% and 7.7%, respectively. Only grade II toxicity was reported. In another study, six patients with borderline or unresectable PC received escalating doses of GEM through intra-splenic infusion of the splenic artery, in 24 h. Median overall survival of 15.3 months was obtained. Unfortunately, 33.35 of the patients developed grade III and IV toxicities including internal bleeding, suggesting the high toxicity of pancreatic perfusion followed by arterial redistribution [224].

Hyperthermic Intraoperative Intraperitoneal Chemotherapy (HIPEC) is used alongside cytoreductive surgeries, to remove tumours that have spread into the abdomen [225]. The procedure is currently being employed as adjuvant therapy after removing a pancreatic tumour to ensure R0 resection. It involves inserting two temperature probes, two inflow catheters into the lower abdomen, and two outflow catheters into the upper abdomen to the perfusion machine, to control the temperature and the flow of the chemotherapy. Temperature probes allow the surgeons to observe the temperature inside the abdomen. After inserting the catheters and the probes, the abdomen is temporarily closed and filled with a saline solution with a temperature of 41–42 °C. Subsequently, chemotherapeutics are added to the solution in two doses over the course of 90 min. After the chemotherapeutics are completely consumed, the abdomen cavity is rinsed with saline solutions. Afterward, the abdomen is reopened for the removal of the machine parts, followed by abdomen suturing [226]. In a recently published prospective study from 2007 to 2018, 39 patients with PC underwent R0 surgical resection followed by GEM administration through HIPEC [226]. The study resulted in a 24% five-year survival rate, and a median survival rate of 17 months. Unfortunately, 59% of patients suffered from recurrence within 13 months, while this treatment was unsuccessful in 10.3% of patients. These results present HIPEC with cytoreductive surgery as possible and safe adjuvant therapy for PC. To further study the feasibility and safety of HIPEC, a phase I clinical trial was initiated in 2021, to compare the effect of HIPEC on cisplatin, Nab-paclitaxel, and GEM, compared to when Nab-paclitaxel and GEM are administrated without the aid of HIPEC, as per Table 4.

Transcatheter arterial radioembolization (TACE) is another form of perfusion therapy that involves using synthetic embolic agents to block the blood vessels supplying tumours, to entrap chemotherapeutics inside tumour. It is considered one of the most effective therapies used in hepatic tumours or metastatic liver tumours arising from other origins such as the pancreas [227,228].

### 3.7. Photodynamic Therapy (PDT)

PDT is based on injecting photosensitizers intravenously, followed by a waiting period, allowing the substance to accumulate in tumour tissues. Subsequently, an endoscopic ultrasound is used to guide an optical fibre into the desired site, followed by tumour illumination for a certain period [229]. PDT induces cell death and microvascular damage in numerous ways, mainly through the production of reactive oxygen species (ROS) which causes damage to multiple cellular structures including the mitochondria and lysosomes [230,231]. Consequently, an acute anti-inflammatory response is induced to enhance the anti-tumour immunity [232]. Moreover, the increased consumption of oxygen creates a hypoxic environment that leads to the overexpression of angiogenic factors and increased angiogenesis [233]. Utilizing PDT in clinical settings to treat PC and other tumours in deep tissues is very challenging, due to the limited depth that the instrument can penetrate and the hypoxic tumour environment [234]. Currently, a phase II VERTPAC-02 clinical trial is studying the efficacy of Ultrasound-Guided Verteporfin PDT for the treatment of advanced PC cancer, as per Table 4.

## 4. Authors’ Opinion on the Future Perspectives of Locoregional Therapies

As discussed throughout the main body of this review, localised interventions are established as very useful adjuvant tools to surgery. By incorporating cytotoxic agents into novel carriers specifically for localised delivery, these agents are retained at a specific tumour site, reducing their systemic drug exposure to minimal toxicity, and maximizing treatment efficacy. However, more scientific and clinical efforts need to be invested in the localised interventions of pancreatic tumours. As discussed throughout this review, this could be manifested by utilizing new advanced materials suitable as carriers for therapeutic agents, or by employing better methods/technologies of administration.

Despite the mentioned benefits of localised therapy, there are several concerns regarding the development of these approaches. Firstly, having a thorough understanding and assessment of the tumour progression and heterogeneity can be challenging. There is a great level of difficulty in developing cell lines that maintain the genetic features of patient-derived xenograft (PDX) models [235]. This modest relationship of cell lines to the cancers from which they had been derived has been established as a major reason for the high failure rate of clinical trials [235]. As a result, scientists should extensively compare the genetics of PC cell lines and the parental tumours to evaluate the extent of genetic drift that occurs during cell line establishment/propagation. In addition to that, more thorough tools assessing the co-relation between in vitro and in vivo studies need to be fabricated.

Secondly, there are several challenges derived from the use of hydrogels for intra-tumoral injection. It has been reported that a majority of injected depots might cause serious inflammation at the site of injection; therefore, the immunogenicity of in situ-forming hydrogels to healthy tissues adjacent to the injected site must be studied carefully [236]. In addition to this, selecting a suitable polymer with high biocompatibility, biodegradability, and safety profile is very challenging, yet it is extremely essential. Hydrogel materials must be biodegradable in tumour microenvironment and not just in hypothetical conditions, degrading at a similar rate to the tumour’s size reduction [236]. However, in the systems where inorganic materials were introduced for the theragnostic purpose, despite the overall reduction in biodegradability of the systems, their long-term stability is greatly enhanced. Therefore, it is the quest of balancing the stability and biodegradability that scientists need to consider when choosing a combination of organic and inorganic compositions for their hydrogels.

In addition, the release behaviour of therapeutic components from a DDS can pose a challenge to the overall performance of the whole system. In the case of nano-formulation DDSs, shape and surface chemistry can play a vital role in the release kinetics of incorporated drugs. For example, Ridolfo et al. reported a 20–30% reduction in the release rate constant of dexamethasone from tubular polymersomes, compared to that of spherical polymersomes [237]. Therefore, morphology and surface interactions need to be thoroughly studied for the optimum design of a delivery system. To enhance the intra-tumoral release of incorporated drugs, carrier systems may be bio-engineered with suitable molecules, enabling the release-on-demand action of the systems, responding to pancreatic tumour microenvironment-specific cues such as hypoxia, pH, enzyme over-expression, or angiogenesis.

Moreover, a recent trend in DDSs is the use of personalised medicine such as three-dimensional printing technology (3DP) due to its many advantages including the ability to tailor the dosage according to each patient’s need with desired release kinetics, its cost-effective benefits, improved patients’ convenience through reducing the frequency of dosing and reducing the burden on the health system. Yi et al. utilised 3D printing to produce a flexible and controlled release polymeric-based patch to release fluorouracil after pancreatic cancer resection [238]. In addition, Talebian et al. developed core/shell 3D printed films, where GEM was enclosed in the dopamine-modified cross-linked alginate hydrogel core, while the shell comprised of cross-linked alginate hydrogel and further coated with polylactic acid (PLA) to provide sustained release of the drug [239]. Despite the interesting findings from these articles, the effort in using 3D to fabricate localised DD is still very much in its infancy. It does, however, help scientists to envisage an advanced method of personalised medication that is effective in the localised intervention of PC. Moreover, based on the aggressive nature of PC and the success of combination therapies, there is significant scope for the development of 3D printed films for localised multimodal chemotherapy. Additionally, one of the major challenges in implementing localised chemotherapeutics is the lack of sufficient data on the exact drug dose required; further studies are needed.

Finally, due to the lack of sufficient randomised controlled trials, and the difficult assessment of local ablative therapies by means of Response Evaluation Criteria in Solid Tumours (RECIST), the specific criteria for introducing locoregional therapy in pancreatic cancer are yet to be established [240]. However, in the past decade, research was directed towards increasing the clinical outcomes of pancreatic cancer patients and providing local control of the disease, by combining locoregional therapies with systemic chemotherapies. As a result, local control of the disease is the main indication of locoregional therapies. For instance, locoregional ablative therapies are introduced with other treatments when the tumour is not suitable for surgery, or in advanced stages for debulking of the tumours rather than complete tumour necrosis [152], and as consolidative treatments in stable diseases. Unfortunately, locally advanced pancreatic cancer is associated with a dismal prognosis; in this case, ablation therapies are introduced as palliative therapies [241]. In addition to this, ablative and embolization therapies are also indicated in pancreatic cancers which have metastasised into other organs, especially the liver. Radiation therapies such as SBRT, on the other hand, are usually introduced after chemotherapy when the cancer is confined to the pancreas.

## 5. Conclusions

PC continues to have a devastating impact on its sufferers, putting health systems under significant strain. To address these issues, it is important that research efforts toward robust and evidence-based research on cancer therapy need to be accelerated. Despite some of the mentioned drawbacks of localised interventions, their benefits outweigh their risk, as discussed throughout this review. With serious investment and efforts in the fabrication of localised interventions, the efficacy of existing therapeutics will be enhanced while their undesirable effects are significantly reduced. This offers all of us, especially cancer sufferers, a great hope toward a better and cancer-free world.

## Figures and Tables

**Table 2:** Injectable DDSs for localised delivery to pancreatic tumours from 2012–2022.

Incorporative Drugs	Type	Polymer	Production Method	Drug Release	Ref.
**GEM** **Montmorillonite (MTT) NPs**	Nanobiohybrid hydrogel	PDLLAPEG-PDLLA, PLEL	Sol-gel of GEM-loaded-MTT NPs	40% after 12 h	[96]
**PTX**	Thermosensitive hydrogel	Poloxamer 407 and Poloxamer 188	Sol-gel	120 h	[97]
**GEM** **Cis platinum (DDP)**	Thermosensitive hydrogel	PDLLAPEG-PDLLA, PLEL	Sol-gel	>10 days	[91]
**GEM**	Adhesive matrix	Styrenated gelatin	-	>80% after 6 h	[108]
**PTX**	Material free DDS	N/A	TRAP depots	32% at 96 h (neutral pH) 60% at 96 h (acidic pH)	[106]
**Ethanol**	Material free DDS	N/A	Multiple injectable needle (MIN)	N/A	[107]

**Table 3:** Implantable DDSs for localised delivery to pancreatic tumours from 2012–2022.

Incorporative Drugs	Type	Polymer	Production Method	Drug Release	Ref.
**GEM**	Membrane	Polyurethane	Solvent casting	Up to 16 days	[112]
**GEM**	Membrane	Polyurethane	Solvent casting	-	[113]
**PTX**	Polymeric drug-embedding matrix	Poly(lactic-co-glycolic)	Dip-Coating	Up to 60 days	[120]
**FOLFIRINOX Combination**	Implantable Iontophoretic Device	Polyurethane	Dip coating	-	[118,119]
**GEM**	Nano-fibre film	Poly (furfuryl methacrylate)	Spin coating	1 h with heat	[129]
**GEM**	Fibrous Scaffolds	Polylactic acid and Hyaluronic acid	Electrospinning	>3 weeks	[125]
**GEM**	Non-woven sheets	Poly (L-lactic acid) (PLLA)	Electrospinning	30 days	[126]
**GEM**	Spun fibres	Alginate or chitosan	Coaxial wet spinning	5–15 days	[130]
**GEM and PTX**	Patch	Polycaprolactone shell and alginate core	Electrospinning	21 days	[128]
**5-FU**	Spun Fibres	PLLA	Electrospinning	30 days	[131]
**FOLFIRINOX Drugs Combination**	Electrospun scaffold	Polyglycolide-co-trimethylene carbonate (PGA-TMC) and porcine gelatin A	Electrospinning	3 weeks	[127]

**Table 4:** List of locoregional therapies in clinical trials. Information was obtained from clinicaltrials.gov [144].

Intervention	Combined Therapy	PC Stage	Trial Phase	Trial Identifier
**Endoscopic Ultrasound (EUS)-Guided Radiofrequency Ablation (RFA)**	Neoadjuvant chemotherapy	Pancreatic ductal adenocarcinoma	Phase II	NCT04990609
	-	PCs	Not Applicable	NCT03218345
	-	Pain relief in PC	Phase IV	NCT04809935
	-	Pancreatic Neuroendocrine Tumours	Not Applicable	NCT04520932
	-	Pancreatic Neuroendocrine Neoplasms	Not Applicable	NCT03834701
**High-intensity focused ultrasound ablation (HIFU)**	FOLFIRINOX regimen	Non-resectable PC	Not Applicable	NCT05262452
	biliary stent	Pancreatic Carcinoma Biliary Obstruction	Not Applicable	NCT03962478
	-	PDAC	Not Applicable	NCT04298242
**Microwave ablation**	Durvalumab 50 MG/ML Tremelimumab GEM	Unresectable Locally Advanced PC	Phase II	NCT04156087
	First-line or second-line chemotherapy	PC Oligohepatic Metastasis	Phase II	NCT04677192
**Cryoablation**	-	PC	Phase I	NCT01335945
**Irreversible electroporation (IRE)**	NK Immunotherapy	Advanced PC	Phase I and II	NCT02718859
	-	Locally advanced PC	-	NCT02841436
	chemotherapy	Unresectable Locally Advanced PC	-	NCT04093141
	GEM	Locally Advanced PC		NCT02981719
	-	Unresectable PC		NCT02041936
	Nivolumab Toll-Like Receptor 9	Metastatic PC	Phase I	NCT04612530
	Nivolumab	Metastatic PC	Phase II	NCT04212026
	-	Locally Advanced PC	-	NCT04276857
	-	PC	-	NCT05170802
	Pembrolizumab	Metastatic PC	Phase II	NCT04835402
	GEM FOLFIRINOX	Advanced Pancreatic Adenocarcinoma	Phase I	NCT03484299
	-	Inoperable Hepatic and Pancreatic Malignancy	-	NCT02822716
	Modified FOLFIRINOX Regimen	Stage III PC	Phase III	NCT03899636
	-	Pancreatic Ductal Adenocarcinoma	-	NCT03257150
**Stereotactic body radiotherapy (SBRT)**	Anti-programmed Cell Death Protein 1(Anti-PD-1)	Late Stage or Recurrent PC Patients	Phase I	NCT03716596
	GEM-based doublets	Advanced PC	Not applicable	NCT02416609
	Modified FOLFIRINOX	Non-metastatic Unresectable Pancreatic	Phase II	NCT03991962
	GEM nab-paclitaxel	Localised PC	Phase II	NCT03492671
	-	Resectable PC	Not applicable	NCT05043857
	Defactinib	Advanced Pancreas Adenocarcinoma	Phase II	NCT04331041
	With or without modified FOLFIRINOX	Locally Advanced Pancreatic Adenocarcinoma		NCT04986930
	-	Pain control for Metastatic PC	Phase II	NCT05114213
	Modified FOLFIRINOX GEM + Nab-paclitaxel GEM + Capecitabine	High Risk and Locally Advanced PC	Phase II	
	Capecitabine Fluorouracil Zoledronic Acid	Locally Advanced PC	Phase II	NCT03073785
	Durvalumab	Pancreatic Adenocarcinoma	Phase I and II	NCT03245541
	Nano-smart AGuIX^®^	Advanced and unresectable Pancreatic Adenocarcinoma	Phase I and II	NCT04789486
	Neoadjuvant GEM plus nab-paclitaxel Neoadjuvant GEM plus nab-paclitaxel with SBRT Neoadjuvant S-1 plus nab-paclitaxel with SBRT	Borderline Resectable PC	Phase II	NCT03777462
	Drug GC4711	borderline resectable and nonresectable PC	Phase IIb	NCT04698915
	Nivolumab CCR2/CCR5 dual antagonist GVAX	Locally Advanced Pancreatic Ductal Adenocarcinoma	Phase I and II	NCT03767582
**Intra-arterial infusion**	GEM nab-paclitaxel using RenovoCath™	Locally Advanced PC	Phase III	NCT03257033
	GEM Oxaliplatin	Locally Advanced PC	Phase II	NCT02635971
